# Semantic Multi-Classifier Systems Identify Predictive Processes in Heart Failure Models across Species

**DOI:** 10.3390/biom8040158

**Published:** 2018-11-26

**Authors:** Ludwig Lausser, Lea Siegle, Wolfgang Rottbauer, Derk Frank, Steffen Just, Hans A. Kestler

**Affiliations:** 1 Institute of Medical Systems Biology, Ulm University, 89069 Ulm, Germany; ludwig.lausser@uni-ulm.de (L.L.); lea.siegle@uni-ulm.de (L.S.); 2 Department of Internal Medicine II, Ulm University, 89081 Ulm, Germany; wolfgang.rottbauer@uniklinik-ulm.de (W.R.); steffen.just@uni-ulm.de (S.J.); 3 Department of Internal Medicine III (Cardiology and Angiology) UKSH, Campus Kiel, 24105 Kiel, Germany; Derk.Frank@uksh.de; 4 DZHK (German Centre for Cardiovascular Research), Partner Site Hamburg/Kiel/Lübeck, 24105 Kiel, Germany

**Keywords:** Heart failure phenotypes, zebrafish, Wistar rat, semantic multi-classifier systems

## Abstract

Genetic model organisms have the potential of removing blind spots from the underlying gene regulatory networks of human diseases. Allowing analyses under experimental conditions they complement the insights gained from observational data. An inevitable requirement for a successful trans-species transfer is an abstract but precise high-level characterization of experimental findings. In this work, we provide a large-scale analysis of seven weak contractility/heart failure genotypes of the model organism zebrafish which all share a weak contractility phenotype. In supervised classification experiments, we screen for discriminative patterns that distinguish between observable phenotypes (homozygous mutant individuals) as well as wild-type (homozygous wild-types) and carriers (heterozygous individuals). As the method of choice we use semantic multi-classifier systems, a knowledge-based approach which constructs hypotheses from a predefined vocabulary of high-level terms (e.g., Kyoto Encyclopedia of Genes and Genomes (KEGG) pathways or Gene Ontology (GO) terms). Evaluating these models leads to a compact description of the underlying processes and guides the screening for new molecular markers of heart failure. Furthermore, we were able to independently corroborate the identified processes in Wistar rats.

## 1. Introduction

Human heart failure (HF) is the leading cause of hospitalization in Western world countries and is associated with high morbidity and mortality thereby putting a large burden on health care costs [1]. Alarmingly, over the last decade, heart failure incidence further increased with a rate of about 1% per year mostly attributed to demographic changes and an aging population [2]. However, up to now, the molecular underpinnings of HF are still only poorly defined but are essential for the development and the clinical implementation of targeted and tailored HF therapies. In this context, the use of established model organisms such as mice and zebrafish to model human heart failure now enable the systematic dissection and definition of the molecular etiology of HF. In recent years, we analyzed several zebrafish lines suffering from heart failure which were identified in large-scale zebrafish forward genetics mutagenesis screens [3]. We characterized these HF zebrafish mutants phenotypically and molecularly, identified the respective underlying genetic defects and verified the relevance in human heart failure [4,5,6,7]. Nevertheless, the molecular networks and common denominators of HF in these mutants are still unknown so far.

Findings from such model organisms can now be generalized via computational approaches by identifying important processes. This augments translational approaches which try to bridge the gap between experimental findings in varying model organisms and to transfer this knowledge to humans. They face the challenge of aligning the behavior of homologous genes and signaling pathways across species. The identification of stable marker signatures in binary or multi-categorial comparisons can identify the most specific markers for a phenotype of interest [8,9,10] and subsequently link it to a biological process. The generalizability of diagnostic models can be used to access the importance of a marker signature [11,12,13]. Nevertheless, in very high-dimensional settings (n≫m, where *n* is the dimensionality and *m* is the cardinality of a dataset) the possibility of reconstructing molecular dependencies in a poorly data-driven way might be limited due to the restricted amount of data. In this case, the training of diagnostic models might be supported by semantic domain knowledge [14,15] on the components of known high-level processes and in turn help to identify them. In this work, we analyze the weak contractility/heart failure phenotype of zebrafish with the help of semantic multi-classifier systems. This model type generates a high-level hypothesis on the underlying processes which is then transferred from one model organism (zebrafish) to another (Wistar rat).

## 2. Results and Discussion

### 2.1. Zebrafish

Weak contractility as a common phenotype of HF is known to be caused by many different genetic mutations [16,17]. Therefore, it can be seen as a diagnostic hypernym comprising a large plethora of genetic backgrounds which lead to a common phenomenon. On a gene expression level, the common structure among these genotypes might be revealed by a common gene signature (i.e., subset of genes) that allows for a highly accurate categorization of all subclasses [10]. It can be seen as a hypothesis on the underlying processes of HF. Interestingly, this analysis does not require identical gene expression levels for all genetic subclasses. Genotype-dependent differences might be taken into account by models that provide individual prototypes for each subclass.

Seven distinct *N*-ethyl-*N*-nitrosourea (ENU)-induced zebrafish mutants all displaying severely reduced contractile performance and finally a HF-like phenotype were subjected to RNA sequencing to define transcriptional mRNA profiles. *Dead beat* mutant embryos suffer from the progressive reduction of cardiac contractility due to a disturbed Vascular endothelial growth factor (VEGF)/Phospholipase C (PLC) γ1 signaling cascade and thereby altered intracellular calcium transients in cardiomyocytes [18]. Mutation of the α1C L-type calcium channel subunit (C-LTCC) leads to fibrillating atria and non-contractile ventricles in *island beat* mutant zebrafish embryos [19]. Furthermore, *main squeeze* and *lost contact* mutant zebrafish embryos develop a HF-like phenotype due to the loss of function of a novel component of the cardiac mechanical stretch sensor, Integrin-linked kinase [5,7]. *Weak atrium* zebrafish mutants display severe cardiac dysfunction due to mutation of atrial myosin heavy chain (myh6) [20], whereas carboxy-terminal truncation of cardiac essential myosin light chain-1 (cmlc1) leads to diminished cardiomyocyte contractility in homozygous mutant *lazy susan* zebrafish embryos [21]. Finally, we also analyzed *tell-tale heart* mutant zebrafish embryos which suffer from contractile dysfunction and a HF-like phenotype due to loss of cardiac myosin light chain 7 (myl7) [4].

In the following, we use semantic multi-classifier systems (S-MCS) for screening for such common gene signatures. These systems are guided by external domain knowledge in form of vocabularies of known gene signatures and processes. The Gene Ontology (GO) and Kyoto Encyclopedia of Genes and Genomes (KEGG) were chosen for our experiments. To take into account effects of multiple genotypes of the *heart failure* phenotype, the one nearest neighbor classifier (1-NN) was used as a multi-centric base classifier. The 1-NN was also used for non-semantic (data-driven) reference experiments. A summary of all experiments can be found in Table 1. They are conducted as 10×10 cross-validation (CV) experiments. Both S-MCS outperform the data-driven 1-NN by at least 17.0% accuracy.

**Figure 1:**
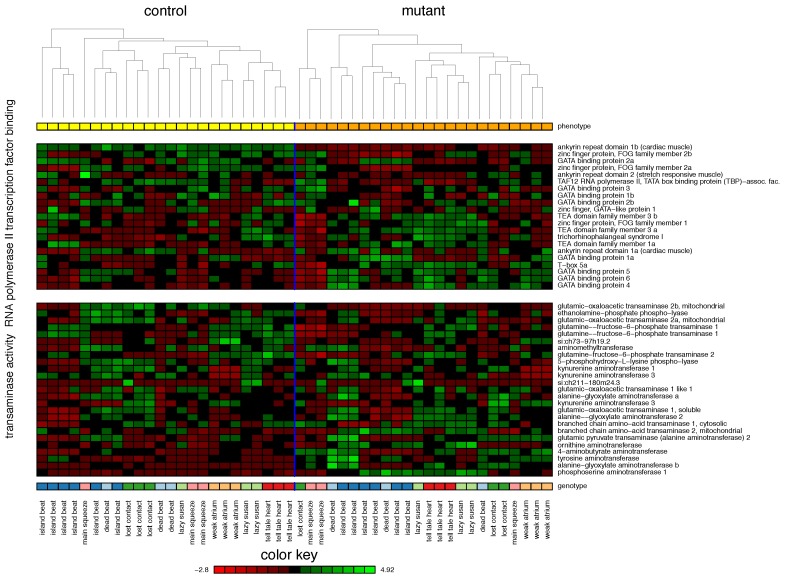
Heatmap of the two most frequently selected GO terms (*RNA polymerase II transcription factor binding* (55.0%) and *transaminase activity* (42.0%)) in zebrafish. The figure provides the gene expression levels of the genes these terms comprise. Within each term, the genes are ordered according their Spearman correlation to the class label (mutant/control). Each gene was *z*-transformed individually. Within each class, hierarchical clustering (average linkage) was used to organize the samples.

The highest classification accuracy was achieved by the S-MCS based on selected GO terms. Mutants and controls were separated with an accuracy of 95.2%. For the mutant class, a sensitivity of 95.8% was reached. The corresponding specificity was 94.6%. Within the top ranking of the five most frequently selected terms already a steep descent in frequency can be observed (39% loss). The two most frequently selected terms were selected in over 50% of all experiments. *RNA polymerase II transcription factor binding* achieved a frequency of 55% followed by *transaminase activity* with a frequency of 42%. The remaining three terms achieve frequencies of 23% (*myoblast differentiation*), 19% (*cellular biogenic amine metabolic process*) and 16% (*hormone activity*). The gene expression levels of the top two GO terms can be found in Figure 1.

The term *RNA polymerase II transcription factor binding* is represented by 21 markers. RNA polymerase II (pol II) is one of three RNA polymerases in eukaryotic cells and responsible for the production of mRNA as well as most microRNA and small nuclear RNA (snRNA). After transcription factors and mediators have been recruited and bound to pol II transcription is initiated at the promotor of the gene. Correct regulation and function of pol II activity is necessary for regular tissue homeostasis. Loss of certain transcription factors such as TEADs (TEF family transcription factors), GATA and FOG (fried of GATA) [22,23,24,25,26,27,28] result in pathophysiologic phenotypes [29] as they are necessary for normal heart development (link to another term found: *myoblast differentiation*). TEADs directly interact with pol II and activate and regulate the expression of several genes involved in cardiac muscle contraction (e.g., MYH6 and MYH7, aka myosin heavy chains α and β [22], SERCA2, aka sarcoendoplasmic reticulum Ca${}^{2+}$-ATPase 2a [24], and others [23]). Loss or overexpression of TEAD1 leads to several phenotypes associated with HF such as fibrosis, contractile dysfunction, hypertrophy, and conduction defects [22,23,24]. DNA binding protein GATA4 and associated proteins (e.g., MEF2a, aka myocyte-specific enhancer factor 2a, and FOG) are involved in cardiac development and are upregulated in cardiac hypertrophy [25,26,27,28,30,31] (again tying in with the term *myoblast differentiation*). Loss of these proteins or their interactions results in phenotypes of cardiac failure including fibrosis, ventricular dilation, contractile dysfunction, hypoxia, and hypertrophy [25,26,27,28,30]. Many more transcription factors such as ankyrin, NELF (negative elongation factor) and SRF (serum response factor) are also involved in maintaining correct cardiac function, and their deregulation results in HF [29,32,33,34]. All in all, this shows that correct function of transcription factors and transcription itself is necessary for healthy heart function.

The RNA-Seq profiles represent the term *transaminase activity* by 25 markers. Transaminases are enzymes which catalyze a reaction to transfer an amine group from an amino acid to a keto acid, where the amine group replaces the keto group. In dilated left ventricles (due to pressure overload) elevated levels of taurine, glutamine, glutamate, aspartate, and alanine have been found compared to undilated heart tissue [35]. This may be due to increased glutamine and alanine metabolism to compensate for anaerobic metabolic stress and energy deficiency in failing hearts [35]. Similarly, ischemic hearts take up more glutamine and aspartate while producing increased levels of alanine in anaplerotic reactions to feed the citric cycle (through transamination) [36]. This provides additional substrates for oxidation and antioxidants in times of stress [36,37]. In this respect, infusion of L-glutamate after ischemic infarction seems to improve cardiac metabolism and reduce infarction size and further tissue damage [38]. This all ties in with a similar pathway found in our analyses of KEGG pathways—*Alanine, aspartate and glutamate metabolism*—reinforcing the importance of amino acid metabolism in the energy metabolism and thus contractility of the heart.

**Figure 2:**
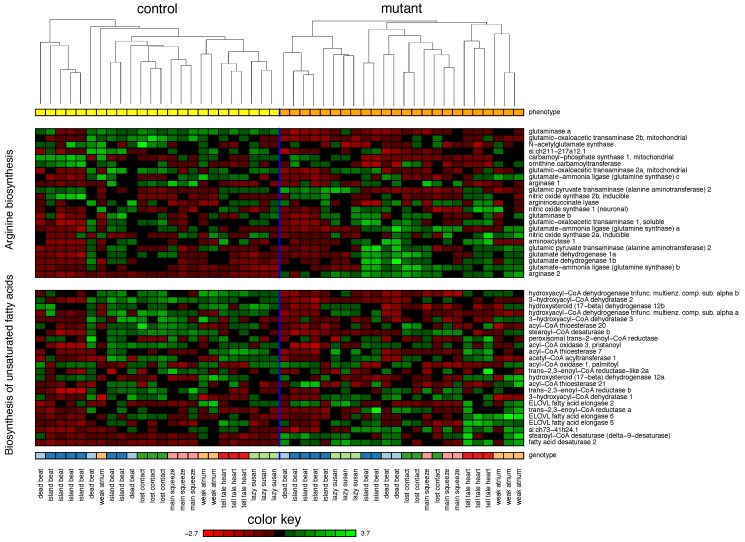
Heatmap of the two most frequently selected KEGG terms *arginine biosynthesis* (75%) and *biosynthesis of unsaturated fatty acids* (55%) in zebrafish. The figure provides the gene expression levels of the genes these terms comprise. Within each term, the genes are ordered according their Spearman correlation to the class label (mutant/control). Each gene was *z*-transformed individually. Within each class, hierarchical clustering (average linkage) was used to organize the samples.

The semantic multi-classifier system based on the KEGG pathway collection achieved an accuracy of 91.5%. The mutant class was detected with sensitivity of 87.5% and a specificity of 95.4%. The five most frequently selected terms are selected in at least 29% of all experiments. The most prominent terms are *arginine biosynthesis* (75%) and *biosynthesis of unsaturated fatty acids* (55%) followed by *fatty acid elongation* (37%), *alanine, aspartate, and glutamate metabolism* (29%) and *cytokine-cytokine receptor interaction* (29%). Figure 2 provides the gene expression levels of the top 2 terms.

*Arginine biosynthesis* is represented by 23 markers. Most arginine is synthesized in the kidneys; however, most cells can synthesize arginine from citrulline [39]. By transaminating glutamine, proline, and ornithine nearly every organ can produce arginine (via citrulline) though this is energetically costly [39] (see *transaminase activity* above). In the cardiovascular system, arginine availability correlates with endothelial function and cardiac contractility [40,41] since arginine is a necessary substrate for nitric oxide (NO) synthesis [41,42,43]. Nitric oxide synthethase 2 is only expressed in cardiac tissue during inflammatory responses such as ischemia, HF and aging [44]. In low doses NO is beneficial for the heart [41,42,44]; however, this effect is eliminated during HF since hypoxia and increased glutamine uptake both inhibit NO production [39,40,45].

The RNA-Seq profiles comprise 24 markers for the term *Biosynthesis of unsaturated fatty acids*. Most of the hearts energy is derived from fatty acid (FA) oxidation [46,47]. Usually, FAs are converted into long-chain acyl-CoA esters, transported into the mitochondria and β-oxidized into acetyl-CoA for the citric cycle [46]. During HF, however, the heart increases glucose metabolism while decreasing FA oxidation leading to reduced energy output and contractility [47]. On the other hand, defects in enzymes of mitochondrial FA synthesis result in a phenotype closely resembling failing or failed heart with dysfunctional mitochondria [48]. Defects in other enzymes of FA synthesis also lead to HF phenotypes with reduced contractility [49,50]. During aging, some unsaturated FAs in mitochondrial cardiolipin are replaced with polyunsaturated FAs which is associated with heart dysfunction and impaired contractility [51]. Overall, this leads to the conclusion that the metabolism of unsaturated FAs plays an important role in cardiac function.

### 2.2. Cross-Species Experiment: Wistar Rats

To validate and prove the cardiac relevance of these findings, we next analyzed transcriptional profiles derived from a cellular rat cardiomyocyte hypertrophy model and compared these cardiomyocyte-specific profiles to our whole zebrafish data.

**Figure 3:**
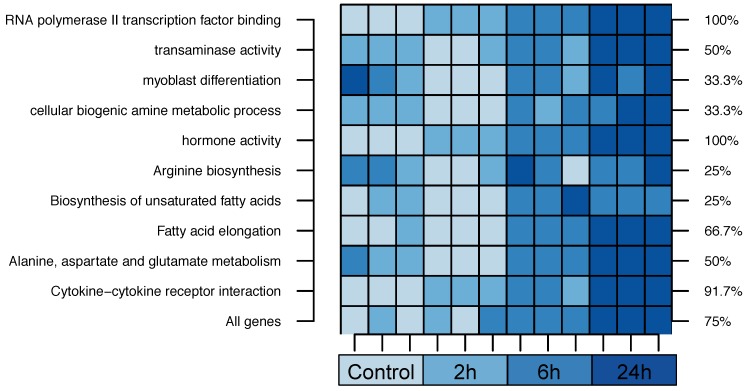
Leave one out cross-validation on rat data using the terms selected in the zebrafish experiments. Additionally, an experiment based on the whole gene expression profiles is shown. Each row indicates a separate experiment. The cell color denotes the predicted stretch time of a sample. The axis on the right gives the overall accuracies (%) of the experiments.

Originally aiming for the identification of new mechanosensitive genes in cardiomyocyte hypertrophy and cardiac remodeling, we subjected neonatal rat ventricular cardiomyocytes (NRVCMs) to cyclic biomechanical stretch for 2, 6, or 24 h (116% at 1 Hz) respectively. RNA isolated from these cells was then analyzed using Agilent’s (Santa Clara, CA, USA) standard Rattus norvegicus 8x60K_60mer mRNA microarrays. The heat map (Figure 3) shows leave one out CV experiments with semantic 1-NN classifiers. Gene expression profiles were thereby restricted to the most frequently selected terms of the *Danio rerio* experiments. As a negative control we also analyzed the prediction based on randomly selected gene sets. Using 1000 repeats of 100 randomly selected genes from the Wistar rat we achieved a mean accuracy of 51.7% (interquartile range, IQR: [41.7–66.7%]).

Biomechanical stretch is a typical inductor of cardiac hypertrophy [52], which in turn represents an integral part of a process termed cardiac remodeling. In the later phases of cardiac remodeling and in addition to hypertrophy, fibrosis and apoptotic cell death take stage and finally lead to contractile dysfunction [53]. Thus, the cardiomyocyte stretch model used here represents an excellent in vitro model to resemble cardiomyocyte hypertrophy and early stages of cardiac remodeling. Interestingly, the high-level processes revealed by our experiments could also be applied to classify this stretch model of rats.

## 3. Conclusions

The major contribution of this work is a large-scale analysis of weak contractility phenotypes observable in the model organism zebrafish. Overall 48 RNA-Seq profiles of seven weak contractility/heart failure genotypes were analyzed. Our knowledge-based approach, a semantic multi-classifier system, allows for the construction of diagnostic models, which can directly be interpreted in high-level terms chosen from a predefined and accepted vocabulary. In our case, the collections of KEGG pathways and GO terms are chosen.

In our study, we use both RNA-Seq and Microarrays in our transfer experiment from zebrafish to rat data. Both platforms can determine gene expression levels, though RNA-Seq is better at quantifying absolute expression levels as well as levels of very low and very high expressing genes [54,55,56,57]. Other than that, both platforms produce comparable results: they are highly reproducible and comparable [58,59,60] and they agree on fold-change direction and significance values [57,61] (though RNA-Seq usually finds more significantly differentially expressed genes [61]). Usually, microarrays are the weaker platform when trying to find differentially expressed genes and determining expression levels. However, RNA-Seq has its own inherent problems. RNA degradation is an issue with both platforms; however, sample preparation for RNA-Seq is more complex the risk for introducing a bias through RNA degradation is higher [55]. In addition, while RNA-Seq has better coverage of all genes (since it is not dependent on probes) it has its own problems with uneven coverage, limiting of sequencing depth and matching genes to the reference sequence [55,57]. Overall, both platforms have their application-specific advantages and drawbacks. For our experimental setup, the arguments usually used in favor of RNA-Seq (better coverage, finding of non-annotated genes, splice variants and low-expressing genes) do not necessarily apply, since we can only use genes which are already annotated and included in a term of the KEGG or GO databases. Since we generate our model on RNA-Seq data and then apply it to microarray data the fine-grained nature of the expression levels is already included in the model. Only coverage might be a problem when transferring the model to microarray data: since microarrays can only evaluate genes for which they have probes, some genes included in the model may not appear in the microarray data. Due to this, some discrepancies may arise when comparing results. This one of the reasons for making the information transfer on a process level. However, the key genes of each pathway are known and probed, so discrepancies should be kept to a minimum. In addition, since we aim for transferability not only between platforms but also between species small discrepancies are expected and out model can clearly withstand them.

Our findings demonstrate the applicability of the terms identified in the lower vertebrate model zebrafish to the mammalian model system rat. As already described, transcriptional profiles were obtained from embryonic zebrafish at 3 days of age. At this stage cardiogenesis, particularly cardiac maturation, is still ongoing although strong rhythmic and sequential contractions of the atrium and the ventricle are well established. Cardiomyocytes isolated from neonatal rat hearts at P1-3 do not represent fully maturated adult cardiomyocytes as well, suggesting that both used model systems are comparable, since their developmental time points and stages almost fully match. In future studies, it will be interesting to compare our findings to adult heart failure models and test their transferability and applicability.

As described before, RNA-Seq profiles were obtained using RNA samples prepared from whole zebrafish embryos at 72 hpf. In contrast to the situation in mammalian cardiovascular disease model systems such as mice, zebrafish embryos at 72 hpf which suffer from severe cardiovascular disorders do usually not exhibit pronounced adverse secondary effects on development and function of other organ systems since e.g., hypoxia is assumed to be only minimal due to the fact that oxygen supply is mostly supplied by passive diffusion from the surrounding water. Nevertheless, recent data derived from transcriptional profiling of the zebrafish mutant *steif* (Unc45b-deficiency) which suffers from severe striated muscle defects including cardiac contractile insufficiency displays the upregulation of genes involved in hypoxia-response [62]. These findings imply that there might be at least a transcriptional hypoxia-like response as a reflection of the cardiac defect, though it is not known if this also reflects cellular hypoxia in vivo. Kajimura and coworker found that hypoxic stress in the developing zebrafish embryo mainly results in embryonic growth retardation [63] which was not observed in the heart failure zebrafish mutants used in our study, suggesting that—if present at all—hypoxia-induced secondary effects are rather minimal here. Whether reduced cardiac contractile performance and compromised blood flow led to other secondary physiological changes which might impact the transcriptional profiles of our zebrafish mutants is yet unknown; however, we also did not see any evidence of such effects.

Nevertheless, cardiac tissue was only a small fraction of the entire tissue mass used for transcriptional profiling, insinuating that some of our zebrafish transcriptional findings might be only associated with non-cardiac tissues and therefore not applicable to HF. To overcome this limitation, bioinformatic evaluation of cardiac- or even cardiomyocyte-specific transcriptional profiles of our heart failure zebrafish mutants will be fundamental. These experiments are already planned for the near future. Additionally, to validate and prove the cardiac relevance of these whole-embryo findings, we also analyzed transcriptional profiles derived from a cellular rat cardiomyocyte hypertrophy model and compared these cardiomyocyte-specific profiles to our whole zebrafish data.

The most frequently selected terms of this analyses, *RNA polymerase II transcription factor binding* (55%, GO) and *transaminase activity* (42%, GO) as well as *arginine biosynthesis* (75%, KEGG) and *biosynthesis of unsaturated FAs* (55%, KEGG) fit well into the context of the weak contractility phenotype. Focusing on the high-level terms also allowed an easy transfer from the model organism *Danio rerio* to the model organism Wistar rat. From a statistical point of view, the selection frequency of the top GO terms and KEGG pathways must be seen as a rare event. They are the only high-level terms out of 152 KEGG pathways and 4354 GO terms with a selection frequency of at least 40%. For both vocabularies, the selection frequency drops to at most 16% within the corresponding top five lists.

In our zebrafish experiments, in comparison to their naive data-driven counterpart, all selection-based approaches improved the CV accuracies. This effect cannot be explained by random dimensionality or feature reduction on their own as shown by the experiments with the one nearest neighbor classifier. Guided selection processes are needed to identify discriminating features. In contrast to other approaches, our system is not focused on individual supportive markers. It also takes into account disadvantageous genes and therefore leads to an assessment of gene sets. While in general the proposed multi-classifier system can operate on random gene sets, the final model will clearly lack the interpretation of known pathways and analyzed gene interactions. Interpretability can only be achieved by incorporating existing domain knowledge in the analysis of complex datasets.

In terms of accuracy, both vocabularies led to comparable results. The semantic multi-classifier systems achieved at least 91.5% CV accuracy. Our study provides a high-level roadmap on the molecular processes on the weak contractility/heart failure phenotype in *Danio rerio* and its corroboration on Wistar rats. This cross-species integration paves the way for a deeper molecular investigation of the involved mechanisms.

## 4. Materials and Methods

### 4.1. Zebrafish Strains, Fractional Shortening Measurements, RNA Isolation and RNA Sequencing

All procedures and experiments in this study were carried out after appropriate institutional approvals (Tierforschungszentrum (TFZ) Ulm University, No. 0183), which conform to the EU Directive 2010/63/EU. Care and breeding of zebrafish (*Danio rerio*) was carried out as described in Kustermann et al. [64]. Fractional shortening was determined by measuring the diameters of the ventricle at the end of contraction (systole) and relaxation (diastole) using the zebraFS software (http://www.benegfx.de) [65]. RNAs from seven different weak contractility/heart failure zebrafish mutant lines were extracted (25 embryos/sample) using the RNeasy Plus Mini Kit (Qiagen, Venlo, The Netherlands) at 72 hpf and subjected to RNA sequencing (llumina HiSeq 2000, Core Facility Genomics, Ulm University, Germany). Samples were collected at 72 hpf since at this specific and early time point cardiac contractile dysfunction was nicely established in all lines without visibly affecting overall embryonic development and morphology.

**Figure 4:**
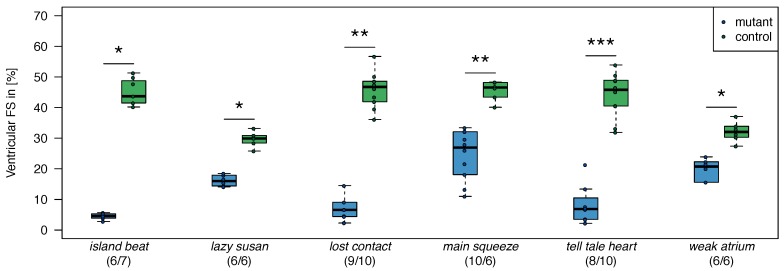
Ventricular fraction shortening of weak contractility/heart failure genotypes (zebrafish). For all genotypes, the differences between mutants and controls were tested (n=7, Wilcoxon-Rank-Sum tests, Bonferroni correction for multiple testing). Significance levels are indicated as *: *p* < 0.05, **: *p* < 0.01, ***: *p* < 0.001.

Table 2 provides an overview on the collected dataset. Overall 48 samples of seven different *weak contractility* heart failure genotypes are available. The dataset splits into 24 pairs of observable mutant phenotypes and the corresponding controls. For each sample, an RNA-Seq profile of 31,953 measurements is recorded. A mutant was categorized as a member of *weak contractility* superclass if it demonstrated significantly reduced ventricular fractional shortening (FS) measurements compared to their wild-type siblings Figure 4; see also [4,5,18,21]):

### 4.2. Stretch Experiments in Neonatal Rat Ventricular Cardiomyocytes (NRVCMs)

Neonatal rat ventricular cardiomyocytes (NRCVMs) were isolated, cultured, and biomechanically stretched as described previously [66]. In brief, NRVCM isolated from 1–3 days old Wistar rats using standard techniques were subjected to cyclic biomechanical stretch for 2, 6, or 24 h using the Flexcell FX-5000T-FLK system (Flexcell international, Dunn Labortechnik, Asbach, Germany). The cells, seeded in a density of 1.5 ×$10^{6}$/well, were stretched on collagen I-coated plates (Bioflex plates, Flexcell international, Dunn Labortechnik) to an extend of 116% at 1 Hz. Non-stretched cells cultivated simultaneously on similarly prepared plates in the same incubator were used as controls. NRVCMs were harvested for RNA isolation using TRIzol (Thermo Fisher Scientific, Waltham, MA, USA) according to the manufacturer’s instructions for use. Desoxyribonuclease I (DNase I) (Sigma-Aldrich, St. Louis, MO, USA) was used to digest potentially contaminating DNA. RNA was further analyzed using Agilent’s standard Rattus norvegicus 8x60K_60mer mRNA microarrays.

### 4.3. Classification

The weak contractility phenotype is analyzed in a binary classification experiment. We are interested in identifying a diagnostic model, a classifier, that allows the distinction between observed mutant phenotype (y=1) or control healthy phenotype (y=0) on a sample-wise level. In this context, each sample will be represented as a feature vector $x={\left(x^{\left(1\right)},\ldots ,x^{\left(n\right)}\right)}^{T}$ and interpreted by the classifier. A diagnostic model will be seen as a function

(1) c:X⟶Y

mapping from the feature space $X\in R^{n}$ to the space of class labels $Y=\left\{0,1\right\}$.

A classifier is adapted for a classification task in a data-driven initial learning phase

(2) $$l:C\times T\mapsto c_{T}.$$

Here, the final classification model $c_{T}$ is chosen from a predefined concept class C and adapted according to a set of labeled training examples $T={\left\{(x_{i},y_{i})\right\}}_{i=1}^{\left|T\right|}$. The subscript ${}_{T}$ will be omitted if not necessary. In a second step, the generalization performance of a trained classifier is estimated on an independent set of test or validation samples $V={\left\{\left(x_{i}^{\prime },y_{i}^{\prime }\right)\right\}}_{i=1}^{\left|V\right|}$. For our experiments the empirical accuracy, sensitivity and specificity are chosen

(3) $${\mathrm{acc}}_{V}=\frac{1}{\left|V\right|}\sum_{(x,y)\in V}I_{\left[c(x)=y\right]},\;{\mathrm{sen}}_{V}=\frac{1}{|V_{1}|}\sum_{\left(x,y\right)\in V_{1}}I_{\left[c(x)=y\right]},\;{\mathrm{spe}}_{V}=\frac{1}{|V_{0}|}\sum_{\left(x,y\right)\in V_{0}}I_{\left[c(x)=y\right]}.$$

Here, $V_{1}$ and $V_{0}$ denote a restriction of V to the samples of class 1 or 0, respectively.

Each binary classification experiment is designed as a 10×10 CV [67]. That is, the overall set of samples S is partitioned into f=10 folds $F_{i}$ of approximately equal size. They are used to generate independent pairs of training sets $T_{i}=S\setminus F_{i}$ and validation sets $V_{i}=F_{i}$ for individual classification experiments. The *i*th classifier $c_{T_{i}}$ is evaluated on $V_{i}$. The average accuracy, sensitivity and specificity are reported. To minimize sampling effects the mean values of r=10 permutations of S are provided. Experiments are performed with the R-package (www.r-project.org) TunePareto [68].

### 4.4. Semantic Multi-Classifier Systems (S-MCS)

We use Semantic Multi-Classifier Systems (S-MCS) for our experiments [14,15]. A S-MCS is a decision ensemble h:X⟶Y that integrates the predictions of several base classifiers

(4) $$h\left(x\right)=u(c_{1}\left(x\right),\ldots ,c_{l}\left(x\right)),$$

where $u:Y_{1}\times \ldots \times Y_{l}⟶Y$ into one final prediction.

**Figure 5:**
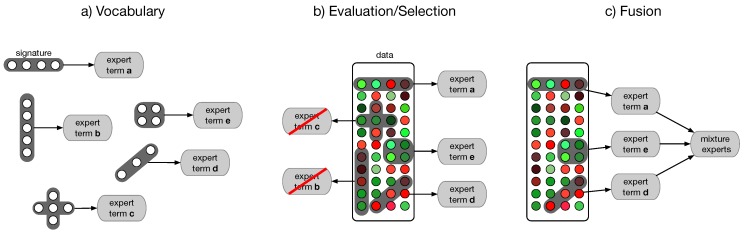
Schematic drawing of a semantic multi-classifier system (S-MCS): The training of a S-MCS uses prior domain knowledge in form of a vocabulary of selected semantic terms (e.g., KEGG pathways of GO terms) for the analysis of incoming gene expression profiles. Each term is analyzed independently by a separate expert (classifier) which focuses on the chosen subset of measurements. The selection of these experts is aggregated via a fusion architecture, leading to a “mixture of experts”.

Each base classifier operates on an individual feature signature $i={\left(i^{\left(1\right)},\ldots ,i^{\left(k\right)}\right)}^{T}$. It is restricted to a limited set of input signals $x^{\left(i\right)}={\left(x^{\left(i^{\left(1\right)}\right)},\ldots ,x^{\left(i^{\left(k\right)}\right)}\right)}^{T}$. Each signature is coupled to a commonly accepted interpretation (term) and chosen from a predefined vocabulary i∈I (Figure 5a). This selection process is based on an internal 3×3 CV on the training set of the corresponding base classifier. The terms with the individual highest accuracies are chosen (Figure 5b). For our experiments we chose an unweighted majority vote as combining scheme and an ensemble size of three base classifiers (Figure 5c). Semantic multi-classifier systems are trained for two vocabularies: the collection of KEGG pathways [69] and the GO terms [70].

### 4.5. Nearest Neighbor Classification

As a base classifier the one nearest neighbor classifier (1-NN) is chosen [71]. The 1-NN is a member of the prototype-based classification *k*-NN algorithms which predict the class label of a query sample v in the following way

(5) $$c\left(v\right)=\underset{y\in Y}{\mathrm{argmax}}\left|\left\{\left(x,y\right)\in {\mathrm{NN}}_{k}\left(v,P\right)\right\}\right|.$$

Here, ${\mathrm{NN}}_{k}\left(v,P\right)$ denotes the *k* nearest neighborhood of v in a set of prototypes $P={\left\{(x_{i},y_{i})\right\}}_{i=1}^{\left|P\right|}$

(6) $${\mathrm{NN}}_{k}\left(v,P\right)=\left\{\left(x,y\right)\;|\;{\mathrm{rk}}_{D_{v}}\left(d(v,x)\right)\leq k\right\}$$

and $D_{v}=\left\{d(v,x)\;|\;(x,y)\in P\right\}$ the set of all pairwise Euclidian distances between v and P. The class label of v is determined via a majority vote of the class labels of the selected candidates. In the case of 1-NN, k=1 and P=T.

### 4.6. Pathways from Kyoto Encyclopedia of Genes and Genomes

The Kyoto Encyclopedia of Genes and Genomes comprises a collection of signaling pathways for a large spectrum of model organisms [69]. For *Danio rerio* 167 signaling pathways are available. For our experiments we restrict ourselves to those pathways that consist of at least 10 components (152 terms).

### 4.7. Gene Ontology Terms

The Gene Ontology is one of the largest attempts to construct an organized and standardized terminology for the categorization of gene products [70]. Its vocabulary is organized in a hierarchical ontology covering three different domains: biological processes, associated cellular components and molecular functions. Most of these terms are linked to manually curated gene lists. We again use those GO terms that comprise at least 10 genes in *Danio rerio* (4354 terms).

## Figures and Tables

**Table 1:** Results of the 10×10 cross-validation experiments (zebrafish). The average accuracy, sensitivity (mutation) and specificity (control) is reported for the semantic multi classifier system (S-MCS) and the 1-nearest neighbor (1-NN) classifier. Additionally, the five most frequently selected KEGG pathways and GO terms are shown (%). The table additionally provides results of experiments with vocabularies comprising 100 sets of randomly selected (with replacement) genes (cardinality per set: 15 or 20). Median average accuracy, sensitivity, and specificity together with interquartile range (IQR) are given.

Cross-validation performance (10×10 cross-validation (CV)):				
		Accuracy(Acc):	Sensitivity(Sens):	Specificity (Spec):
S-MCS (GO)		95.2%	95.8%	94.6%
S-MCS (KEGG)		91.5%	87.5%	95.4%
1-NN (all genes)		74.8%	79.2%	70.4%
**Most Frequent GO Terms (%):**				
1. RNA Polymerase II Transcription Factor Binding				55%
2. Transaminase Activity				42%
3. Myoblast Differentiation				23%
4. Cellular Biogenic Amine Metabolic Process				19%
5. Hormone Activity				16%
**Most Frequent KEGG Pathways (%):**			
1. Arginine biosynthesis				75%
2. Biosynthesis of unsaturated fatty acids				55%
3. Fatty acid elongation				37%
4. Alanine, aspartate and glutamate metabolism				29%
5. Cytokine-cytokine receptor interaction				29%
**Random Vocabularies (100 repetitions, 10×10 cross-validation (CV)):**				
		Acc:	Sens:	Spec:
S-MCS (100 × 15 rand. genes)	median	91.6%	91.7%	92.9%
	IQR	[88.8–94.6%]	[87.1–94.6%]	[88.3–95.5%]
S-MCS (100 × 20 rand. genes)	median	92.6%	91.3%	93.5%
	IQR	[89.1–95.4%]	[87.8–95.0%]	[91.2–96.7%]
1-NN (100 rand. genes)	median	78.5%	78.5%	78.8%
	IQR	[67.2–86.1%]	[66.6–87.5%]	[70.0–87.2%]

**Table 2:** Overview of the analyzed dataset: The dataset comprises gene expression profiles of 48 whole-fish samples seven individual genotypes (rows) with a weak contractility. The genotypes are named according to their observable heartbeat. For each genotype, the number of samples (mutants(mut)/controls(crt)) is reported.

No.	Genotype	Samples (mut/crt)
1.	*dead beat* (m582)	(3/3)
2.	*island beat* (m458)	(6/6)
3.	*lazy susan* (m647)	(3/3)
4.	*lost contact* (hu801)	(3/3)
5.	*main squeeze* (m347)	(3/3)
6.	*tell-tale heart* (m225)	(3/3)
7.	*weak atrium* (m229)	(3/3)
	summary	(24/24)
